# Three Decades of Living Liver Donor Outcomes: A National Cohort Analysis of Patterns in Mortality and Recovery

**DOI:** 10.1155/joot/2545887

**Published:** 2026-10-04

**Authors:** Morgan E. Kelly, Tianna M. Truby, Jennifer C. Lai, Sharad I. Wadhwani

**Affiliations:** ^1^ Department of Pediatrics University of California San Francisco San Francisco California USA ucsf.edu; ^2^ Department of Medicine University of California San Francisco San Francisco California USA ucsf.edu

**Keywords:** liver transplantation, living donors, mortality, patient safety, postoperative complications, registries, risk assessment

## Abstract

Living donor liver transplantation offers significant benefits to recipients, yet risks to donors remain incompletely characterized. We examined short‐ and intermediate‐term outcomes associated with living liver donation using the Scientific Registry of Transplant Recipients. This retrospective cohort study included 8619 donors aged 18–65 years who donated between 1989 and 2023, of whom 8314 had follow‐up data for secondary outcomes. Donors were categorized by era: early (1989–2006) and contemporary (2007–2023). Primary outcomes were 90‐day and 2‐year mortality; secondary outcomes were changes in physical capacity, functional status, and employment. Mean donor age was 36 years, and 53% were female. Eight donors (0.1%) died within 90 days and 18 (0.2%) within 2 years. Mortality was lower in the contemporary era at both 90 days (HR: 0.14, 95% CI: 0.03–0.70) and 2 years (HR: 0.06, 95% CI: 0.02–0.18). Reduced physical capacity at 6 months was more common among contemporary‐era donors (aOR: 3.25, 95% CI: 1.32–8.00) and Hispanic donors (aOR: 2.07, 95% CI: 1.29–3.32). Reduced employment was more common among non‐White donors at 6 months and 1 year (aOR: 2.04, 95% CI: 1.40–2.97 at 1 year), and among female donors at all timepoints (aOR: 1.71, 95% CI: 1.29–2.25 at 1 year). Living liver donor mortality has declined over the past three decades, and long‐term physical impairments are uncommon. Reduced employment following donation is disproportionately reported by female and non‐White donors. These findings provide a benchmark for center‐level assessment of donor outcomes and may inform donor counseling that addresses occupational circumstances alongside surgical risk.

**Summary**
• Practitioner points
1. Donor mortality following living liver donation declined approximately 85% over the past three decades and is now 0.04% within 90 days of surgery.
2. Long‐term physical impairments are uncommon, with fewer than 2% of donors reporting reduced physical capacity at 1‐year postdonation.

## 1. Introduction

Liver transplantation is the definitive treatment for patients with acute liver failure and end‐stage liver disease; however, persistent shortages of deceased donor organs result in prolonged wait times and ongoing waitlist mortality [1–3]. Roughly one in four adults listed for liver transplantation dies on the waitlist or experiences clinical deterioration precluding transplant, and although children represent only a small minority of listings, approximately 5% of listed children die or become too ill to undergo transplant [1, 2]. Living donor liver transplantation (LDLT) offers significant survival benefit to recipients, yet remains underutilized in the United States [3, 4]. LDLT is especially critical for children, who experience significantly higher waitlist mortality rates compared to adults, with infants under 1 year having the highest waitlist mortality of any age group [5].

While the benefits of LDLT to recipients have been well described, concerns remain regarding potential short‐ and long‐term physical and psychological risks to living liver donors [6–9]. Living liver donation involves a complex hepatectomy in healthy individuals, and thus raises important ethical, medical, and psychosocial considerations [10]. Donor safety and concerns about adverse medical outcomes have been identified as primary barriers to pursuing LDLT [11]. While previous estimates of donor mortality range between 0.1% and 0.5%, these estimates are largely derived from single‐center studies or international cohorts, which may not reflect contemporary outcomes within the United States [12–14]. A recent meta‐analysis provided updated global estimates, but data specific to the United States remain limited. Understanding precise and current estimates of donor risks for LDLT is critical to informing potential donors [8, 10].

Using data from the Scientific Registry of Transplant Recipients (SRTR), we examined trends in perioperative morbidity and mortality among living liver donors over the past three decades and assessed whether specific donor groups faced increased risks. To our knowledge, this represents the first national SRTR‐based analysis focused specifically on mortality and outcomes among living liver donors in the United States. We hypothesized that donor outcomes have improved over time and that LDLT has become safer in the contemporary era.

## 2. Methods

This study used data from the SRTR. The SRTR data system includes data on all donors, wait‐listed candidates, and transplant recipients in the United States, submitted by the members of the Organ Procurement and Transplantation Network (OPTN). The Health Resources and Services Administration (HRSA), US Department of Health and Human Services, provides oversight to the activities of the OPTN and SRTR contractors. This study was deemed exempt from IRB oversight by the University of California San Francisco IRB (reference number 20‐31241); therefore, no informed consent was required or obtained.

### 2.1. Participant Sample

We identified all living liver donors aged 18 –65 at the time of donation between November 27, 1989, and March 2, 2023 (*N* = 8981). We excluded 362 donors (4%) who had neither a documented death date nor a patient status date, as neither an event date nor a censoring date could be determined. This yielded a primary analytic cohort of 8619 for mortality analyses. Of these donors, 8314 (97%) had at least one postdonation follow‐up record and comprised the analytic cohort for secondary outcomes (Supporting Figure 1). Missingness among covariates was minimal. Race was missing for 2 donors (0.02%), and these donors were excluded from the multivariable models. Region was missing for 304 donors (4%) but was not included in any regression model and therefore did not affect model sample size.

### 2.2. Primary Exposure

The primary exposure in this study was era of donation. We stratified our sample based on date of donation into early (1989–2006) and contemporary (2007–2023) eras, with 2007 selected to mark the transition from center‐level discretion in donor care to a nationally standardized framework for donor selection, protection, and outcome surveillance. In 2007, the Centers for Medicare & Medicaid Services established the first federal requirements for the selection and protection of living donors [15]. That same year, OPTN/UNOS began requiring living donor programs to maintain written protocols across all phases of donation and extended mandatory follow‐up reporting from one to two years postdonation [16].

### 2.3. Outcomes

Our primary outcomes were mortality within 90 days of donation and overall mortality within 2 years following the date of transplant, both modeled as time‐to‐event outcomes. Both mortality outcomes were all‐cause. Deaths from any cause were counted, as cause of death was not recorded consistently in SRTR for living donors and was not available for analysis. Secondary outcomes included changes in physical capacity, functional status, and employment status at 6 months, 1 year, and 2 years postdonation. All secondary outcomes were ascertained through patient‐reported survey data submitted by transplant centers to the OPTN and available in the SRTR Standard Analysis Files.

Functional status was assessed by comparing each donor’s predonation and postdonation reports of ability to perform activities of daily living (ADLs). We classified donors as having functional decline if they reported normal functional status before donation and required assistance with ADLs postdonation. All other patterns (stable normal function, stable impairment, or improvement in performing ADLs) were classified as no decline.

Physical capacity was assessed by comparing predonation and postdonation reports of mobility. We classified donors as having decline in physical capacity if they reported no mobility limitations predonation and any mobility limitations postdonation. All other patterns (stable normal physical capacity, stable limitations, or improvement in mobility) were classified as no decline.

Employment status was assessed by comparing predonation and postdonation work status. We classified donors as having a reduction in employment if they reported working before donation and not working postdonation. We classified all other patterns (continued employment, continued unemployment, or return to work) as no change in employment.

### 2.4. Covariates

We included donor age group (18–40 or 41–65), donor sex, donor race, and donor ethnicity, as well as recipient age group (pediatric or adult) at time of transplantation, as covariates. These covariates were selected a priori based on their potential to confound the association between era and postdonation outcomes. Donor race and ethnicity were recorded in SRTR as reported by transplant programs.

### 2.5. Missing Data

All analyses used a complete‐case approach. No imputation was performed for missing outcomes, follow‐up records, or covariates. Availability of individual secondary outcome variables varied by outcome and timepoint. Physical capacity, functional status, and employment status were analyzed separately using outcome‐ and timepoint‐specific complete cases. Missing data for one outcome or timepoint did not exclude a donor from analyses of other available outcomes. We evaluated patterns of missingness by comparing baseline characteristics between donors with and without available outcome data (Supporting Table 1).

### 2.6. Statistical Analysis

Donor and recipient characteristics were summarized using frequencies and percentages for categorical variables and compared using Pearson chi‐square tests. Fisher’s exact test with Monte Carlo simulation was used when expected cell counts were less than 5. Cox proportional hazards models were used to evaluate differences in postdonation mortality across eras. Survival time was calculated from the date of donation to the date of death or the last known follow‐up date. Donors with a documented death date were classified as having experienced the event, and those without a documented death date were right‐censored at the patient status date. Donors missing both a death date and patient status date were excluded because survival analysis follow‐up could not be calculated. Adjusted Cox models included time era, donor age group, recipient age group, donor race, and donor sex. Results are reported as hazard ratios with 95% confidence intervals.

Secondary outcomes were evaluated using separate logistic regression models for each outcome and timepoint, with odds ratios greater than 1 indicating greater odds of decline from baseline. Unadjusted estimates were obtained from univariable models containing the listed predictor only. Adjusted estimates were obtained from multivariable models including time era, donor age group, recipient age group, donor sex, donor race, and donor ethnicity. Donor ethnicity was included in the secondary outcome models, where the greater number of events permitted a larger adjustment set. At the 2‐year timepoint, secondary outcome data were available only for contemporary‐era donors. Thus, time era was omitted from these models, and 2‐year estimates apply to the contemporary cohort alone. Differences in the magnitude of odds ratios across timepoints were interpreted descriptively, given models were fitted separately at each timepoint.

All tests were two‐sided with α = 0.05. Analyses were conducted using R Version 4.5.1 (R Core Team, 2025).

## 3. Results

### 3.1. Data Availability and Missingness

Of 8981 eligible donors, 362 (4%) lacked both a documented death date and patient status date and were excluded from mortality analyses, resulting in a primary analytic cohort of 8619 donors. Of these, 8314 (97%) had available follow‐up data and comprised the secondary analytic cohort. Availability of secondary‐outcome data declined over follow‐up, with 60%–67% of donors having evaluable data at 6 months and 37%–39% at 2 years. Patterns of data availability differed by several baseline characteristics and are detailed in Supporting Table 1.

### 3.2. Cohort Characteristics

Table 1 presents data on the demographic characteristics of the living liver donors in our sample (*N* = 8314), stratified by era of donation. The mean age of the donors at time of donation was 36 years. The majority of the donors identified as White and non‐Hispanic. Donors from the contemporary era were more likely to be female and donate to an adult recipient compared to donors from the early era.

**TABLE 1:** Characteristics of living donors.

Characteristics	Overall (*N* = 8314)	1989–2006 (*N* = 2683)	2007–2023 (*N* = 5631)	*p* value
Mean age at donation (SD)	36.4 (10)	36.0 (10)	36.6 (10)	0.004
Sex, *n* (%)				
Female	4404 (53%)	1339 (50%)	3065 (54%)	< 0.001
Male	3910 (47%)	1344 (50%)	2566 (46%)	
Race, *n* (%)				0.329
Non‐White	761 (9%)	233 (9%)	528 (9%)	
White	7551 (91%)	2448 (91%)	5103 (91%)	
Missing	2 (0%)	2 (0%)	0 (0%)	
Ethnicity, *n* (%)				0.217
Non‐Hispanic	7260 (87%)	2325 (87%)	4935 (88%)	
Hispanic	1054 (13%)	358 (13%)	696 (12%)	
Region				< 0.001
Foreign country	15 (0%)	7 (0%)	8 (0%)	
North Central	1622 (20%)	496 (19%)	1126 (20%)	
Northeast	2659 (32.0%)	923 (34%)	1736 (31%)	
South	1952 (24%)	540 (20%)	1412 (25%)	
West	1824 (22%)	626 (23%)	1198 (21%)	
Unknown	242 (3%)	91 (3%)	151 (3%)	
Recipient age group, *n* (%)				< 0.001
Adult	6671 (80%)	2047 (76%)	4624 (82%)	
Pediatric	1643 (20%)	636 (24%)	1007 (18%)	

*Note:* This table presents data on the demographic characteristics of the secondary analytic cohort, stratified by era of donation.

### 3.3. Primary Outcomes

Over the 34‐year study period, 8 (0.1%) donors died within 90 days of donation, and 83 (1%) total deaths were recorded, with 18 deaths occurring within 2 years of donation (0.2%) (Figure 1). Since only eight deaths occurred within 90 days, 90‐day mortality was evaluated using univariable Cox proportional hazards models only. Donors who underwent surgery in the contemporary era had a lower hazard of 90‐day mortality compared to those who underwent surgery in the early era (HR = 0.14, 95% CI: 0.03,0.70, *p* value = 0.02). There was no statistically significant difference in 90‐day mortality risk by donor age (18–40 years vs. 41–65 years, *p* value = 0.855), donor sex (*p* value = 0.867), donor race (White vs. non‐white, *p* value = 0.745), or recipient age (pediatric vs. adult, *p* value = 0.673) (Table 2). Multivariable analysis was not performed due to the limited number of events observed (*n* = 8).

**FIGURE 1:**
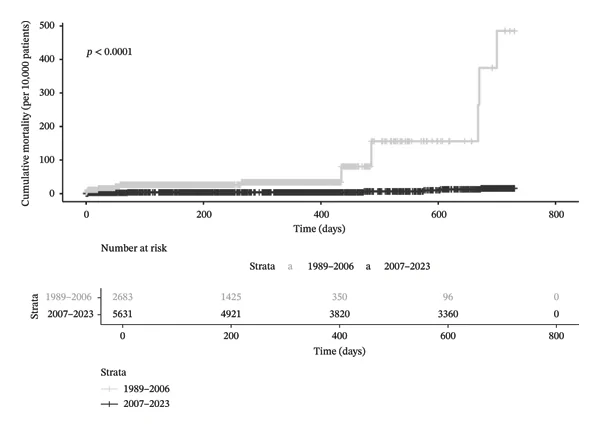
Cumulative postoperative mortality by era. Cumulative mortality rate per 10,000 donors, stratified by donation era. Kaplan–Meier estimates are shown with follow‐up censored at 2 years. Numbers at risk are displayed below the X‐axis.

**TABLE 2:** Univariable Cox proportional hazards models for 90‐day mortality.

Variables	Univariable HR (95% CI)	Univariable *p* value
Time era (2007–2023 vs. 1989–2006)	0.14 (0.03, 0.70)	0.016
Donor age (41–65 vs. 18–40)	1.14 (0.27, 4.78)	0.855
Recipient age (pediatric vs. adult)	1.41 (0.28, 6.99)	0.673
Sex (female vs. male)	0.89 (0.22, 3.55)	0.867
Race (non‐White vs. White)	1.42 (0.17, 11.51)	0.745

*Note:* Hazard ratios and corresponding *p* values are shown for 90‐day mortality by donation era, donor age, recipient age, donor sex, and donor race. Italic terms indicate the reference category for each variable.

In univariable analysis, donors in the contemporary era had a lower hazard of two‐year mortality compared to the early era (HR = 0.04, 95% CI: 0.01, 0.14, *p* value < 0.001). Donors aged 41–65 years had a higher risk of two‐year mortality compared to donors aged 18–40 years (HR 3.66, 95% CI: 1.37, 9.76, *p* value = 0.009). There was no statistically significant difference in two‐year mortality risk by donor sex (*p* value = 0.085), donor race (*p* value = 0.584), or recipient age (*p* value = 0.42).

For 2‐year mortality, the adjusted model included time era, donor age, recipient age, donor race, and donor sex. Donor ethnicity and region were considered for inclusion in the multivariable Cox proportional hazards model, but excluded due to perfect separation, which prevented model convergence. Donors in the contemporary era had a lower hazard of two‐year mortality compared to those in the early era (HR = 0.06, 95% CI: 0.02, 0.18, *p* value < 0.001). Predictors of 90‐day and two‐year mortality are summarized in Figure 2. Donors aged 41–65 years had a higher risk of two‐year mortality compared to those aged 18–40 years (HR = 3.08, 95% CI: 1.12, 8.50, *p* value = 0.03). Female sex trended toward a lower hazard of death, though this did not reach statistical significance (HR = 0.50, 95% CI: 0.19, 1.36, *p* value = 0.18). There was no statistically significant difference in two‐year mortality by recipient age (*p* value = 0.649) or donor race (*p* value = 0.834), which persists from univariable analysis (Table 3).

**FIGURE 2:**
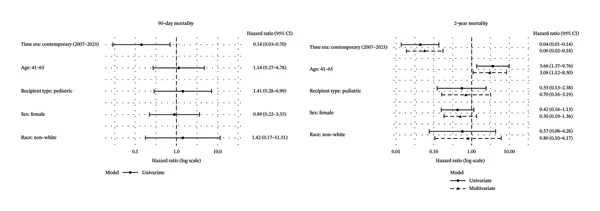
Forest plots for 90‐day and 2‐year mortality. Forest plots of hazard ratios with 95% confidence intervals for predictors of 90‐day (left) and 2‐year (right) mortality. Variables include donation era, donor age, recipient age, donor sex, and donor race. Univariable estimates (circles) are shown for both outcomes; multivariable estimates (triangles) are shown for 2‐year mortality only.

**TABLE 3:** Univariable and multivariable Cox proportional hazards models for 2‐year mortality.

Variables	Univariable HR (95% CI)	Univariable *p* value	Multivariable HR (95% CI)	Multivariable *p* value
Time era (2007–2023 vs. 1989–2006)	0.04 (0.01, 0.14)	< 0.001	0.06 (0.02, 0.18)	< 0.001
Donor age (41–65 vs. 18–40)	3.66 (1.37, 9.76)	0.009	3.08 (1.12, 8.50)	0.030
Recipient age (pediatric vs. adult)	0.55 (0.13, 2.38)	0.420	0.70 (0.16, 3.19)	0.649
Sex (female vs. male)	0.42 (0.16, 1.13)	0.085	0.50 (0.19, 1.36)	0.175
Race (non‐White vs. White)	0.57 (0.08, 4.28)	0.584	0.80 (0.10, 6.17)	0.834

*Note:* Hazard ratios and corresponding *p* values from univariable and multivariable analyses of 2‐year mortality by donation era, donor age, recipient age, donor sex, and donor race. Italic terms indicate the reference category for each variable.

### 3.4. Secondary Outcomes

A decline in physical capacity was reported by two percent (*n* = 110) of donors at 6 months postdonation, decreasing to one percent (*n* = 64) at 1 year, which remained stable at 2 years postdonation (1%, *n* = 45). Fourteen percent (*n* = 774) of donors reported reduced function at 6 months postdonation, which decreased to nine percent (*n* = 461) at 1 year and eight percent (*n* = 245) at 2 years postdonation. Six percent (*n* = 313) of donors reported a decrease in employment status from baseline at 6 months postdonation; this proportion decreased to five percent (*n* = 238) by 1 year and increased slightly at 2 years postdonation (6%, *n* = 187). Availability of secondary‐outcome data decreased over follow‐up across all three outcomes, ranging from 60% to 67% at 6 months, 54%–60% at 1 year, and 37%–39% at 2 years (Supporting Table 2).

#### 3.4.1. Physical Capacity

At 6 months postdonation, donors who underwent surgery in the contemporary era were roughly three times more likely to experience a decrease in physical capacity compared to those who underwent surgery in the early era (aOR = 3.25, 95% CI: 1.32, 8.00, *p* value = 0.010). This pattern was not statistically significant at 1 year (aOR = 1.36, 95% CI: 0.58, 3.19, *p* value = 0.473). Early‐era donors had no available 2‐year follow‐up, so the association between time era and physical capacity could not be estimated at this timepoint. The 2‐year estimates given as follows apply to contemporary‐era donors only. Donors aged 41–65 years experienced increased odds of a decrease in physical capacity postdonation compared to donors aged 18–40 years, demonstrating a consistent trend across all timepoints; however, statistical significance was only observed at the 1‐year follow‐up (aOR = 2.20, 95% CI: 1.32, 3.68, *p* value = 0.002).

Changes in physical capacity did not differ significantly between donors with a pediatric recipient and donors with an adult recipient at 6 months or 1‐year postdonation. However, at 2 years, donors with pediatric recipients in the contemporary era were less likely to report decreased physical capacity (aOR = 0.2, 95% CI: 0.05, 0.83, *p* value = 0.027).

Non‐White donors had roughly twice the odds of decreased physical capacity at 1‐year postdonation (aOR = 2.27, 95% CI: 1.09, 4.71, *p* value = 0.028), but did not show any significant association at 6 months or 2 years. Hispanic donors were more likely to report decreased physical capacity compared to non‐Hispanic donors at 6 months (aOR = 2.07, 95% CI: 1.29, 3.32, *p* value = 0.003) and at 1 year postdonation (aOR = 2.88, 95% CI: 1.60, 5.18, *p* value < 0.001). At 2 years, only contemporary‐era donors had available data, and the association was not significant in this subgroup (aOR = 1.94, 95% CI: 0.92, 4.12, *p* value = 0.083) (Supporting Table 3).

#### 3.4.2. Functional Status

Donor functional status did not differ significantly by era at 6 months (aOR = 0.93, 95% CI: 0.74, 1.16, *p* value = 0.504) or at 1 year postdonation (aOR = 0.88, 95% CI: 0.66, 1.16, *p* value = 0.365); however, we observed a trend toward lower odds of experiencing a decline in functional status among donors in the contemporary era compared to the early era. Donors in the early era were lost to follow‐up by the 2‐year timepoint, preventing further comparison. The 2‐year estimates given as follows apply to contemporary‐era donors only. Older donor age (41–65 years vs. 18–40 years) was associated with increased odds of reporting functional decline at 6 months (aOR = 1.20, 95% CI: 1.02, 1.40, *p* value = 0.029), 1 year (aOR = 1.27, 95% CI: 1.04, 1.55, *p* value = 0.022), and 2 years (aOR = 1.51, 95% CI: 1.15, 1.99, *p* value = 0.003). Donation to pediatric recipients (vs. adult recipients) was associated with decreased odds of functional decline at all timepoints: 6 months (aOR = 0.64, 95% CI: 0.52, 0.80, *p* value < 0.001), 1 year (aOR = 0.50, 95% CI: 0.37, 0.68, *p* value < 0.001), and 2 years (aOR = 0.45, 95% CI: 0.29, 0.70, *p* value < 0.001). Donor sex was not significantly associated with changes in functional status at any timepoint. Non‐White race was associated with increased odds of functional decline across all timepoints (6 months aOR = 1.48, 95% CI: 1.15, 1.89, *p* value = 0.002; 1 year aOR = 1.39, 95% CI: 1.01, 1.93, *p* value = 0.044; 2 year aOR = 1.82, 95% CI: 1.22, 2.70, *p* value = 0.003). Hispanic ethnicity was associated with increased odds of functional decline at 6 months (aOR = 1.46, 95% CI: 1.18, 1.81, *p* value < 0.001) and at 2 years (aOR = 1.70, 95% CI: 1.19, 2.45, *p* value = 0.004), but not at 1 year (aOR = 1.26, 95% CI: 0.95, 1.67, *p* value = 0.101) (Supporting Table 4).

#### 3.4.3. Employment Status

Employment status did not differ significantly by era at 6 months (aOR = 0.81, 95% CI: 0.56, 1.17, *p* value = 0.262) or 1‐year postdonation (aOR = 1.11, 95% CI: 0.68, 1.80, *p* value = 0.676). Time‐era differences could not be evaluated at 2 years, as employment data were only available for contemporary‐era donors at this timepoint. Donor age and recipient age were not associated with postdonation employment outcomes at any timepoint. Female donors had higher odds of reporting a reduction in employment status compared to male donors at 6 months (aOR = 1.32, 95% CI: 1.04, 1.67, *p* value = 0.020), 1 year (aOR = 1.71, 95% CI: 1.29, 2.25, *p* value < 0.001), and 2 years (aOR = 1.69, 95% CI: 1.24, 2.31, *p* value = 0.001). Non‐White donors had higher odds of reporting a reduction in employment at 6 months (aOR = 1.45, 95% CI: 1.02–2.08, *p* value = 0.040) and 1 year (aOR = 2.04, 95% CI: 1.40, 2.97, *p* value < 0.001), though this association was not significant at 2 years (aOR = 1.41, 95% CI: 0.89, 2.22, *p* value = 0.141) (Supporting Table 5). Hispanic ethnicity was not significantly associated with employment outcomes at any timepoint.

## 4. Discussion

To our knowledge, this is the largest national analysis of postoperative outcomes among living liver donors in the United States, and the first to examine mortality alongside patient‐reported physical, functional, and employment outcomes. We found that 90‐day mortality declined from 0.26% in the early era to 0.04% in the contemporary era, which represents a relative reduction of approximately 85%. The reduction in 90‐day mortality suggests improved procedural safety, as mortality attributable to the operation itself would be expected to occur in the immediate postoperative period. This finding is consistent with advancements in surgical techniques, perioperative management, and donor selection criteria [6, 10, 17, 18]. Two‐year mortality also declined across eras (adjusted HR = 0.06, 95% CI: 0.02, 0.18); however, cause of death was not available for most donors, and deaths occurring months to years after donation are unlikely to be procedure‐related. Loss to follow‐up was substantially greater in the early era, reducing the number at risk over the two‐year interval. The era difference at two years may therefore reflect changes in population health or differential follow‐up rather than changes in donor management.

We found that postoperative declines in functional status and physical capacity generally improved over time, with only a small percentage reporting ongoing limitations by 1‐year postdonation. Contemporary‐era donors reported diminished physical capacity more often at 6 months postdonation. This pattern did not persist at one year, which suggests these declines were temporary. These findings are consistent with prior reports of donor recovery. Reviews of donor quality of life have described most physical and psychosocial measures returning to baseline within the first year postdonation [8, 9]. Our estimates of reduced physical capacity fall at the lower end of previously reported ranges, which may be due to ascertainment differences between center‐reported items to SRTR and dedicated quality of life questionnaires.

Donors to pediatric recipients reported functional decline less often at every timepoint. This may reflect the smaller graft volume required for a pediatric recipient that would require a less extensive hepatectomy; however, we did not have data on graft type or resection extent and could not test this directly. Female donors had higher odds of reduced employment at six months, one year, and two years, and non‐White donors had higher odds of reduced employment at six months and one year. These associations were not accompanied by corresponding differences in functional status or physical capacity by sex, which argues against a solely physiological explanation. It is plausible that women in the United States are more likely to assume unpaid caregiving responsibilities during a recipient’s recovery, particularly when the recipient is a family member. Non‐White donors may be more likely to have hourly or physically demanding work in which extended medical leave is less easily accommodated. SRTR data do not capture caregiving obligations, occupation, or leave benefits, so we could not test these explanations. The observed differences in employment status by sex and race indicate the need to better understand how social and demographic factors influence donor recovery, particularly as they relate to employment outcomes.

These findings have practical implications for donor counseling, clinical practice, and quality improvement. We provide United States–specific estimates of donor mortality to replace the single‐center and international figures currently used to inform consent discussions. Perioperative mortality in the contemporary era is approximately 4 per 10,000 donors, roughly 2% of living liver donors report reduced physical capacity at 6 months postdonation, and reports of limitation become markedly less frequent by one year. These national estimates provide a benchmark against which individual centers can compare their own donor outcomes. Counseling should extend beyond surgical risk to include the possibility of delayed return to work. The persistence of reduced employment among female and non‐White donors identifies a target for intervention and provides a basis for better characterizing psychosocial and demographic factors that impact donor outcomes. Programs could incorporate assessment of occupational circumstances into donor evaluation and connect donors at higher risk of income disruption with available support. These implications also support the expansion of living liver donation by ensuring that prospective donors receive counseling with current, nationally representative data and that those most likely to experience reduced employment are identified before donation.

This study should be interpreted in the context of the following limitations. We excluded 362 donors (4%) from mortality analyses due to lacking both a death date and a patient status date, and 305 donors (4%) from secondary analyses for lacking any postdonation follow‐up record. Both groups were disproportionately from the early era, so complete‐case analyses may have introduced selection bias (Supporting Table 1). Availability of secondary outcome data also declined over follow‐up, with only 37% –39% of the secondary cohort having data at 2 years and no early‐era observations at that timepoint, which limits interpretation of longer‐term comparisons between eras. Cause of death was not recorded for most donors, so we report all‐cause mortality and cannot distinguish between procedure‐related and background mortality. We did not compare observed donor mortality with that expected in an age‐ and sex‐matched general population. Such a comparison was outside the scope of this analysis, which characterized trends in donor outcomes over time rather than benchmarking donor mortality against population norms. Future work should evaluate whether mortality rates differ substantially from the general population. We did not link donor outcomes to recipient graft or survival outcomes, which may shape donor recovery, particularly when the donor is also a caregiver. Examining paired donor–recipient outcomes is an important direction for future work. Finally, standard limitations to administrative dataset studies apply, including data completeness. However, SRTR’s mandatory capture of all transplants in the United States ensures this analysis provides the largest national assessment of living liver donor outcomes to date.

## 5. Conclusion

In this national cohort of living liver donors in the United States, mortality declined significantly over the past three decades, and long‐term postoperative impairments were infrequent. Reductions in employment were more common among female and non‐White donors and persisted beyond the period of physical recovery, indicating that donor evaluation and follow‐up should address occupational and financial circumstances alongside surgical risk. These findings provide current, nationally representative estimates to demonstrate the safety of LDLT, inform donor counseling, and support center‐level assessment of donor outcomes.

## Funding

This study was supported by the National Institute of Diabetes and Digestive and Kidney Diseases (Grant nos. P30DK026743, K23DK132454, and T32DK060414) and the National Institute on Aging (Grant nos. K24AG080021 and P30DK026743) (support for SIW, JCL).

## Disclosure

The data reported here have been supplied by the Hennepin Healthcare Research Institute (HHRI) as the contractor for the Scientific Registry of Transplant Recipients (SRTR). The interpretation and reporting of these data are the responsibility of the authors and in no way should be seen as an official policy of or interpretation by the SRTR or the US Government. A preliminary version of this work was presented as an abstract at the World Transplant Congress, San Francisco, CA, August 2025, and published in abstract form in the *American Journal of Transplantation* (Am J Transplant. 2025; 25[8 Suppl 1]).

## Conflicts of Interest

The authors declare no conflicts of interest.

## Supporting Information

Additional supporting information can be found online in the Supporting Information section.

## Supporting information


**Supporting Information** The following supporting information is available for this article. Supporting Table 1. Baseline characteristics of donors with and without available primary outcome data, comparing the 362 donors excluded for missing survival information with the 8,619 donors retained in mortality analyses. Supporting Table 2. Availability and distribution of physical capacity, functional status, and employment data at 6 months, 1 year, and 2 years postdonation among the secondary analytic cohort. Supporting Table 3. Unadjusted and adjusted logistic regression models for decreased physical capacity at 6 months, 1 year, and 2 years postdonation. Supporting Table 4. Unadjusted and adjusted logistic regression models for decreased functional status at 6 months, 1 year, and 2 years postdonation. Supporting Table 5. Unadjusted and adjusted logistic regression models for decreased employment status at 6 months, 1 year, and 2 years postdonation. Supporting Figure 1. Cohort selection diagram showing inclusion and exclusion criteria, from the 8,981 eligible living liver donors through the primary analytic cohort for mortality analyses (*n* = 8,619) and the secondary analytic cohort for postdonation outcomes (*n* = 8,314).

## Data Availability

The data that support the findings of this study are available from the Scientific Registry of Transplant Recipients. Restrictions apply to the availability of these data, which were used under license for this study. Data are available from https://www.srtr.org/requesting-data/data-requests/with the permission of the Scientific Registry of Transplant Recipients.
