# Supplementary material for: Three Decades of Living Liver Donor Outcomes: A National Cohort Analysis of Patterns in Mortality and Recovery
Source: J Transplant. 2026 Oct 4;2026:2545887. doi: 10.1155/joot/2545887 (PMC13635687; doi:10.1155/joot/2545887)
Supplement: Supplementary file 1 [file JOOT-2026-2545887-s001.docx]

**Supplemental Table 1: Comparison of characteristics of donors with and without available primary outcome data**

|  | **Excluded** **(N=362)** | **Included** **(N=8619)** | **Overall** **(N=8981)** | **P-value** |
| --- | --- | --- | --- | --- |
| **Time Era** |  |  |  |  |
| 1989-2006 | 315 (87.0%) | 2929 (34.0%) | 3244 (36.1%) | <0.001 |
| 2007-2023 | 47 (13.0%) | 5690 (66.0%) | 5737 (63.9%) |  |
| **Donor Age** |  |  |  |  |
| 18-40 | 296 (81.8%) | 5733 (66.5%) | 6029 (67.1%) | <0.001 |
| 41–65 | 66 (18.2%) | 2886 (33.5%) | 2952 (32.9%) |  |
| **Sex** |  |  |  |  |
| Male | 162 (44.8%) | 4046 (46.9%) | 4208 (46.9%) | 0.715 |
| Female | 200 (55.2%) | 4573 (53.1%) | 4773 (53.1%) |  |
| **Race** |  |  |  |  |
| Non-White | 48 (13.3%) | 797 (9.2%) | 845 (9.4%) | 0.038 |
| White | 314 (86.7%) | 7820 (90.7%) | 8134 (90.6%) |  |
| Missing | 0 (0%) | 2 (0.0%) | 2 (0.0%) |  |
| **Ethnicity** |  |  |  |  |
| Non-Hispanic | 309 (85.4%) | 7534 (87.4%) | 7843 (87.3%) | 0.516 |
| Hispanic | 53 (14.6%) | 1085 (12.6%) | 1138 (12.7%) |  |
| **Recipient Age** |  |  |  |  |
| Adult Recipient | 102 (28.2%) | 6786 (78.7%) | 6888 (76.7%) | <0.001 |
| Pediatric Recipient | 260 (71.8%) | 1833 (21.3%) | 2093 (23.3%) |  |
| **Region** |  |  |  |  |
| Foreign Country | 5 (1.4%) | 18 (0.2%) | 23 (0.3%) | <0.001 |
| North Central | 72 (19.9%) | 1692 (19.6%) | 1764 (19.6%) |  |
| Northeast | 85 (23.5%) | 2694 (31.3%) | 2779 (30.9%) |  |
| South | 66 (18.2%) | 2054 (23.8%) | 2120 (23.6%) |  |
| Unknown | 59 (16.3%) | 304 (3.5%) | 363 (4.0%) |  |
| West | 75 (20.7%) | 1857 (21.5%) | 1932 (21.5%) |  |

- Values are presented as n(%). P-values were calculated using chi-square tests. Fisher’s exact test with Monte Carlo simulation was used when any expected cell count was less than 5. Missing primary outcome data were defined as the absence of both a documented death date and a patient status date (DFL_PX_STAT_DT)
- Donors recorded as Latino ethnicity in SRTR are referred to as Hispanic

**Supplemental Table 2: Availability and Distribution of Secondary Outcome Data by Follow-up Assessment Among Donors in the Secondary Analytic Cohort**

|  | **Physical Capacity** | | | |
| --- | --- | --- | --- | --- |
|  | **Missing n (%)** | **Available n (%)** | **Decrease n (%)** | **No Change n (%)** |
| **6 months** | 2722 (32.7%) | 5592 (67.3%) | 110 (2.0%) | 5482 (98.0%) |
| **1 year** | 3333 (40.1%) | 4981 (59.9%) | 64 (1.3%) | 4917 (98.7%) |
| **2 years** | 5089 (61.2%) | 3225 (38.8%) | 45 (1.4%) | 3180 (98.6%) |
|  | **Functional status** | | | |
|  | **Missing n (%)** | **Available n (%)** | **Decrease n (%)** | **No Change n (%)** |
| **6 months** | 2777 (33.4%) | 5537 (66.6%) | 774 (14.0%) | 4763 (86.0%) |
| **1 year** | 3386 (40.7%) | 4928 (59.3%) | 461 (9.4%) | 4467 (90.6%) |
| **2 years** | 5129 (61.7%) | 3185 (38.3%) | 245 (7.7%) | 2940 (92.3%) |
|  | **Employment Status** | | | |
|  | **Missing n (%)** | **Available n (%)** | **Decrease n (%)** | **No Change n (%)** |
| **6 months** | 3322 (40.0%) | 4992 (60.0%) | 313 (6.3%) | 4679 (93.7%) |
| **1 year** | 3796 (45.7%) | 4518 (54.3%) | 238 (5.3%) | 4280 (94.7%) |
| **2 years** | 5203 (62.6%) | 3111 (37.4%) | 187 (6.0%) | 2924 (94.0%) |

- Missing and available percentages were calculated using the secondary analytic cohort (n=8314).
- Decrease and no change percentages were calculated among the donors with available data for the applicable outcome and timepoint.
- Each secondary outcome represented change relative to baseline and was dichotimized as decrease or no change. Outcome data availability was evaluated separately for physical capacity, functional status, and employment status at each timepoint. Missing data from one outcome or timepoint did not exclude a donor from otherwise eligible analysis. No imputation was performed for missing outcomes.

**Supplemental Table 3: Logistic Regression Models for Decreased Physical Capacity from Baseline**

|  | **6-month Assessment** | | | | | |
| --- | --- | --- | --- | --- | --- | --- |
|  | **Unadjusted** | | | **Adjusted** | | |
|  | **OR** | **95% CI** | **P-value** | **OR** | **95% CI** | **P-value** |
| **Time Era** |  |  |  |  |  |  |
| 1989–2006 | REF |  |  | REF |  |  |
| 2007–2023 | 3.28 | 1.33, 8.06 | 0.010 | 3.25 | 1.32, 8.00 | 0.010 |
| **Donor Age** |  |  |  |  |  |  |
| 18–40 | REF |  |  | REF |  |  |
| 41–65 | 1.35 | 0.92, 1.98 | 0.127 | 1.45 | 0.98, 2.15 | 0.064 |
| **Recipient Age** |  |  |  |  |  |  |
| Adult Recipient | REF |  |  | REF |  |  |
| Pediatric Recipient | 0.73 | 0.43, 1.25 | 0.251 | 0.74 | 0.43, 1.27 | 0.272 |
| **Sex** |  |  |  |  |  |  |
| Male | REF |  |  | REF |  |  |
| Female | 0.77 | 0.53, 1.13 | 0.186 | 0.74 | 0.51, 1.09 | 0.129 |
| **Race** |  |  |  |  |  |  |
| White | REF |  |  | REF |  |  |
| Non-White | 1.21 | 0.66, 2.23 | 0.530 | 1.38 | 0.75, 2.56 | 0.302 |
| **Ethnicity** |  |  |  |  |  |  |
| Non-Hispanic | REF |  |  | REF |  |  |
| Hispanic | 1.87 | 1.17, 2.98 | 0.009 | 2.07 | 1.29, 3.32 | 0.003 |
|  | **1-year Assessment** | | | | | |
|  | **Unadjusted** | | | **Adjusted** | | |
|  | **OR** | **95% CI** | **P-value** | **OR** | **95% CI** | **P-value** |
| **Time Era** |  |  |  |  |  |  |
| 1989–2006 | REF |  |  | REF |  |  |
| 2007–2023 | 1.40 | 0.60, 3.25 | 0.438 | 1.36 | 0.58, 3.19 | 0.473 |
| **Donor Age** |  |  |  |  |  |  |
| 18–40 | REF |  |  | REF |  |  |
| 41–65 | 1.94 | 1.18, 3.18 | 0.009 | 2.20 | 1.32, 3.68 | 0.002 |
| **Recipient Age** |  |  |  |  |  |  |
| Adult Recipient | REF |  |  | REF |  |  |
| Pediatric Recipient | 0.54 | 0.24, 1.18 | 0.122 | 0.54 | 0.24, 1.21 | 0.137 |
| **Sex** |  |  |  |  |  |  |
| Male | REF |  |  | REF |  |  |
| Female | 0.77 | 0.47, 1.26 | 0.298 | 0.72 | 0.44, 1.19 | 0.198 |
| **Race** |  |  |  |  |  |  |
| White | REF |  |  | REF |  |  |
| Non-White | 1.67 | 0.82, 3.40 | 0.158 | 2.27 | 1.09, 4.71 | 0.028 |
| **Ethnicity** |  |  |  |  |  |  |
| Non-Hispanic | REF |  |  | REF |  |  |
| Hispanic | 2.29 | 1.29, 4.06 | 0.004 | 2.88 | 1.60, 5.18 | < 0.001 |
|  | **2-year Assessment** | | | | | |
|  | **Unadjusted** |  |  | **Adjusted** |  |  |
|  | **OR** | **95% CI** | **P-value** | **OR** | **95% CI** | **P-value** |
| **Time Era** |  |  |  |  |  |  |
| 1989-2006 | — | — | — | — | — | — |
| 2007–2023 | — | — | — | — | — | — |
| **Donor Age** |  |  |  |  |  |  |
| 18–40 | REF |  |  | REF |  |  |
| 41–65 | 1.22 | 0.67, 2.22 | 0.523 | 1.16 | 0.62, 2.14 | 0.644 |
| **Recipient Age** |  |  |  |  |  |  |
| Adult Recipient | REF |  |  | REF |  |  |
| Pediatric Recipient | 0.20 | 0.05, 0.84 | 0.028 | 0.20 | 0.05, 0.83 | 0.027 |
| **Sex** |  |  |  |  |  |  |
| Male | REF |  |  | REF |  |  |
| Female | 1.11 | 0.61, 2.01 | 0.735 | 1.12 | 0.61, 2.04 | 0.713 |
| **Race** |  |  |  |  |  |  |
| White | REF |  |  | REF |  |  |
| Non-White | 0.89 | 0.32, 2.51 | 0.831 | 1.14 | 0.40, 3.24 | 0.803 |
| **Ethnicity** |  |  |  |  |  |  |
| Non-Hispanic | REF |  |  | REF |  |  |
| Hispanic | 1.80 | 0.86, 3.76 | 0.119 | 1.94 | 0.92, 4.12 | 0.083 |

- Each outcome was defined relative to the donor’s baseline status and dichotomized as decrease, coded as 1, vs no change coded as 0. Separate logistic regression models were fitted for each outcome and follow-up assessment. Unadjusted estimates were obtained from univariable models containing the listed predictor only. Adjusted estimates were obtained from a multivariable model, including time era, donor age group, recipient age group, donor sex, race and ethnicity. Analyses used outcome specific complete cases. No imputation was performed.
- Physical capacity data for the 2-year assessment were available for donors from the 2007-2023 era. Therefore, the association between the time era and decreased physical capacity could not be estimated at this assessment. The reported estimates evaluate characteristics within the contemporary era cohort. OR, odds ratio; CI, Confidence Interval

**Supplemental Table 4: Logistic Regression Models for Decreased Functional Status from Baseline**

|  | **6-month Assessment** | | | | | |
| --- | --- | --- | --- | --- | --- | --- |
|  | **Unadjusted** | | | **Adjusted** | | |
|  | **OR** | **95% CI** | **P-value** | **OR** | **95% CI** | **P-value** |
| **Time Era** |  |  |  |  |  |  |
| 1989–2006 | REF |  |  | REF |  |  |
| 2007–2023 | 0.94 | 0.75, 1.17 | 0.570 | 0.93 | 0.74, 1.16 | 0.504 |
| **Donor Age** |  |  |  |  |  |  |
| 18–40 | REF |  |  | REF |  |  |
| 41–65 | 1.20 | 1.03, 1.40 | 0.023 | 1.20 | 1.02, 1.40 | 0.029 |
| **Recipient Age** |  |  |  |  |  |  |
| Adult Recipient | REF |  |  | REF |  |  |
| Pediatric Recipient | 0.66 | 0.53, 0.82 | < 0.001 | 0.64 | 0.52, 0.80 | < 0.001 |
| **Sex** |  |  |  |  |  |  |
| Male | REF |  |  | REF |  |  |
| Female | 0.99 | 0.85, 1.15 | 0.917 | 0.98 | 0.84, 1.15 | 0.845 |
| **Race** |  |  |  |  |  |  |
| White | REF |  |  | REF |  |  |
| Non-White | 1.30 | 1.02, 1.65 | 0.037 | 1.48 | 1.15, 1.89 | 0.002 |
| **Ethnicity** |  |  |  |  |  |  |
| Non-Hispanic | REF |  |  | REF |  |  |
| Hispanic | 1.35 | 1.09, 1.67 | 0.006 | 1.46 | 1.18, 1.81 | < 0.001 |
|  | **1-year Assessment** | | | | | |
|  | **Unadjusted** | | | **Adjusted** | | |
|  | **OR** | **95% CI** | **P-value** | **OR** | **95% CI** | **P-value** |
| **Time Era** |  |  |  |  |  |  |
| 1989–2006 | REF |  |  | REF |  |  |
| 2007–2023 | 0.88 | 0.67, 1.16 | 0.369 | 0.88 | 0.66, 1.16 | 0.365 |
| **Donor Age** |  |  |  |  |  |  |
| 18–40 | REF |  |  | REF |  |  |
| 41–65 | 1.32 | 1.08, 1.60 | 0.006 | 1.27 | 1.04, 1.55 | 0.022 |
| **Recipient Age** |  |  |  |  |  |  |
| Adult Recipient | REF |  |  | REF |  |  |
| Pediatric Recipient | 0.50 | 0.37, 0.68 | < 0.001 | 0.50 | 0.37, 0.68 | < 0.001 |
| **Sex** |  |  |  |  |  |  |
| Male | REF |  |  | REF |  |  |
| Female | 1.03 | 0.85, 1.26 | 0.730 | 1.04 | 0.85, 1.26 | 0.727 |
| **Race** |  |  |  |  |  |  |
| White | REF |  |  | REF |  |  |
| Non-White | 1.17 | 0.85, 1.61 | 0.325 | 1.39 | 1.01, 1.93 | 0.044 |
| **Ethnicity** |  |  |  |  |  |  |
| Non-Hispanic | REF |  |  | REF |  |  |
| Hispanic | 1.15 | 0.87, 1.51 | 0.329 | 1.26 | 0.95, 1.67 | 0.101 |
|  | **2-year Assessment** | | | | | |
|  | **Unadjusted** |  |  | **Adjusted** |  |  |
|  | **OR** | **95% CI** | **P-value** | **OR** | **95% CI** | **P-value** |
| **Time Era** |  |  |  |  |  |  |
| 1989–2006 | — | — | — | — | — | — |
| 2007–2023 | — | — | — | — | — | — |
| **Donor Age** |  |  |  |  |  |  |
| 18–40 | REF |  |  | REF |  |  |
| 41–65 | 1.50 | 1.15, 1.95 | 0.002 | 1.51 | 1.15, 1.99 | 0.003 |
| **Recipient Age** |  |  |  |  |  |  |
| Adult Recipient | REF |  |  | REF |  |  |
| Pediatric Recipient | 0.45 | 0.29, 0.70 | < 0.001 | 0.45 | 0.29, 0.70 | < 0.001 |
| **Sex** |  |  |  |  |  |  |
| Male | REF |  |  | REF |  |  |
| Female | 1.02 | 0.79, 1.33 | 0.861 | 1.03 | 0.79, 1.34 | 0.855 |
| **Race** |  |  |  |  |  |  |
| White | REF |  |  | REF |  |  |
| Non-White | 1.46 | 0.99, 2.15 | 0.055 | 1.82 | 1.22, 2.70 | 0.003 |
| **Ethnicity** |  |  |  |  |  |  |
| Non-Hispanic | REF |  |  | REF |  |  |
| Hispanic | 1.48 | 1.04, 2.10 | 0.030 | 1.70 | 1.19, 2.45 | 0.004 |

- Each outcome was defined relative to the donor’s baseline status and dichotomized as decrease, coded as 1, vs no change coded as 0. Separate logistic regression models were fitted for each outcome and follow-up assessment. Unadjusted estimates were obtained from univariable models containing the listed predictor only. Adjusted estimates were obtained from a multivariable model, including time era, donor age group, recipient age group, donor sex, race and ethnicity. Analyses used outcome specific complete cases. No imputation was performed.
- Functional status data for the 2-year assessment were available for donors from the 2007-2023 era. Therefore, the association between the time era and decreased functional status could not be estimated at this assessment. The reported estimates evaluate characteristics within the contemporary era cohort. OR, odds ratio; CI, Confidence Interval

**Supplemental Table 5: Logistic Regression Models for Decreased Employment Status from Baseline**

|  | **6-month Assessment** | | | | | |
| --- | --- | --- | --- | --- | --- | --- |
|  | **Unadjusted** | | | **Adjusted** | | |
|  | **OR** | **95% CI** | **P-value** | **OR** | **95% CI** | **P-value** |
| **Time Era** |  |  |  |  |  |  |
| 1989–2006 | REF |  |  | REF |  |  |
| 2007–2023 | 0.82 | 0.57, 1.18 | 0.294 | 0.81 | 0.56, 1.17 | 0.262 |
| **Donor Age** |  |  |  |  |  |  |
| 18–40 | REF |  |  | REF |  |  |
| 41–65 | 0.85 | 0.67, 1.09 | 0.205 | 0.88 | 0.68, 1.13 | 0.303 |
| **Recipient Age** |  |  |  |  |  |  |
| Adult Recipient | REF |  |  | REF |  |  |
| Pediatric Recipient | 1.20 | 0.90, 1.58 | 0.211 | 1.09 | 0.82, 1.46 | 0.552 |
| **Sex** |  |  |  |  |  |  |
| Male | REF |  |  | REF |  |  |
| Female | 1.32 | 1.04, 1.66 | 0.021 | 1.32 | 1.04, 1.67 | 0.020 |
| **Race** |  |  |  |  |  |  |
| White | REF |  |  | REF |  |  |
| Non-White | 1.46 | 1.03, 2.07 | 0.035 | 1.45 | 1.02, 2.08 | 0.040 |
| **Ethnicity** |  |  |  |  |  |  |
| Non-Hispanic | REF |  |  | REF |  |  |
| Hispanic | 1.27 | 0.92, 1.74 | 0.149 | 1.24 | 0.90, 1.71 | 0.189 |
|  | **1-year Assessment** | | | | | |
|  | **Unadjusted** | | | **Adjusted** | | |
|  | **OR** | **95% CI** | **P-value** | **OR** | **95% CI** | **P-value** |
| **Time Era** |  |  |  |  |  |  |
| 1989–2006 | REF |  |  | REF |  |  |
| 2007–2023 | 1.16 | 0.72, 1.88 | 0.540 | 1.11 | 0.68, 1.80 | 0.676 |
| **Donor Age** |  |  |  |  |  |  |
| 18–40 | REF |  |  | REF |  |  |
| 41–65 | 0.82 | 0.61, 1.08 | 0.158 | 0.84 | 0.62, 1.12 | 0.227 |
| **Recipient Age** |  |  |  |  |  |  |
| Adult Recipient | REF |  |  | REF |  |  |
| Pediatric Recipient | 1.09 | 0.78, 1.52 | 0.608 | 0.93 | 0.66, 1.31 | 0.679 |
| **Sex** |  |  |  |  |  |  |
| Male | REF |  |  | REF |  |  |
| Female | 1.68 | 1.27, 2.21 | < 0.001 | 1.71 | 1.29, 2.25 | < 0.001 |
| **Race** |  |  |  |  |  |  |
| White | REF |  |  | REF |  |  |
| Non-White | 1.99 | 1.38, 2.87 | < 0.001 | 2.04 | 1.40, 2.97 | < 0.001 |
| **Ethnicity** |  |  |  |  |  |  |
| Non-Hispanic | REF |  |  | REF |  |  |
| Hispanic | 1.29 | 0.91, 1.84 | 0.154 | 1.29 | 0.90, 1.85 | 0.168 |
|  | **2-year Assessment** | | | | | |
|  | **Unadjusted** |  |  | **Adjusted** |  |  |
|  | **OR** | **95% CI** | **P-value** | **OR** | **95% CI** | **P-value** |
| **Time Era** |  |  |  |  |  |  |
| 1989–2006 | — | — | — | — | — | — |
| 2007–2023 | — | — | — | — | — | — |
| **Donor Age** |  |  |  |  |  |  |
| 18–40 | REF |  |  | REF |  |  |
| 41–65 | 0.82 | 0.60, 1.13 | 0.224 | 0.82 | 0.59, 1.13 | 0.225 |
| **Recipient Age** |  |  |  |  |  |  |
| Adult Recipient | REF |  |  | REF |  |  |
| Pediatric Recipient | 1.07 | 0.73, 1.56 | 0.729 | 0.96 | 0.65, 1.42 | 0.843 |
| **Sex** |  |  |  |  |  |  |
| Male | REF |  |  | REF |  |  |
| Female | 1.66 | 1.22, 2.27 | 0.001 | 1.69 | 1.24, 2.31 | 0.001 |
| **Race** |  |  |  |  |  |  |
| White | REF |  |  | REF |  |  |
| Non-White | 1.40 | 0.90, 2.19 | 0.138 | 1.41 | 0.89, 2.22 | 0.141 |
| **Ethnicity** |  |  |  |  |  |  |
| Non-Hispanic | REF |  |  | REF |  |  |
| Hispanic | 1.06 | 0.68, 1.65 | 0.790 | 1.02 | 0.65, 1.60 | 0.918 |

- Each outcome was defined relative to the donor’s baseline status and dichotomized as decrease, coded as 1, vs no change coded as 0. Separate logistic regression models were fitted for each outcome and follow-up assessment. Unadjusted estimates were obtained from univariable models containing the listed predictor only. Adjusted estimates were obtained from a multivariable model, including time era, donor age group, recipient age group, donor sex, race and ethnicity. Analyses used outcome specific complete cases. No imputation was performed.
- Working status data for the 2-year assessment were available for donors from the 2007-2023 era. Therefore, the association between the time era and decreased working status could not be estimated at this assessment. The reported estimates evaluate characteristics within the contemporary era cohort. OR, odds ratio; CI, Confidence Interval

**Supplemental Figure 1**. Consort-style diagram outlining cohort selection with inclusion and exclusion criteria.
